# Uptake and Use of Biologic Therapies in Paediatric Immune‐Mediated Inflammatory Diseases: An Australian Population‐Based Study

**DOI:** 10.1002/pds.70412

**Published:** 2026-07-01

**Authors:** Jun Ni Ho, Jodie Hillen, Natasha Nassar, Helga Zoega, Timothy Beukelman, Nicole Pratt, Benjamin Daniels

**Affiliations:** ^1^ Quality Use of Medicines and Pharmacy Research Centre, College of Health Adelaide University Adelaide Australia; ^2^ School of Pharmacy and Pharmaceutical Sciences, Faculty of Health, Medicine and Behavioural Sciences The University of Queensland Queensland Australia; ^3^ Child Population and Translational Health Research, Children's Hospital at Westmead Clinical School, Faculty of Medicine and Health The University of Sydney Sydney Australia; ^4^ Charles Perkins Centre The University of Sydney Sydney Australia; ^5^ Leeder Centre for Health Policy, Economics and Data, Faculty of Medicine and Health The University of Sydney Sydney Australia; ^6^ Centre of Public Health Sciences, Faculty of Medicine University of Iceland Reykjavik Iceland; ^7^ University of Alabama at Birmingham Birmingham Alabama USA; ^8^ Medicines Intelligence Research Program, School of Population Health University of New South Wales Sydney Australia

**Keywords:** biologic medicines, immune‐mediated inflammatory diseases, medicine utilisation, paediatric population, trends, uptake

## Abstract

**Purpose:**

To quantify the real‐world use and describe characteristics of paediatric patients initiating biologic medicines for immune‐mediated inflammatory diseases (IMIDs).

**Methods:**

Dispensing claims for Australians aged less than 18 years were used to examine uptake and use trends of nine biologic medicines used for the treatment of IMIDs. A 15% random sample of the population was used for dispensings between 2013 and 2017, and a 30% sample for dispensings between 2017 and 2020. Incidence and prevalence rates of use per 1 000 000 population per year were calculated. Patient demographics at the time of first dispensing were determined and the number of unique medicines dispensed per person across the study period was calculated.

**Results:**

Incidence of use of biologics increased from 108 per million in 2014 to 192 per million in 2020, while prevalence increased from 352 per million to 877 per million over the same period. 1131 children were dispensed at least one biologic medicine; infliximab, adalimumab or etanercept accounted for 80% of dispensings. Mean age at first dispensing was 14 years (standard deviation: 3 years) and 51% were male. Of children initiated on biologics, 55% were first dispensed treatment for inflammatory bowel diseases and 29% for inflammatory arthropathies. While most children were dispensed only one unique biologic medicine, 15% were dispensed two, and 3% three or more over the study period.

**Conclusions:**

These results suggest that children remain on treatment for extended periods of time and nearly one in five children were dispensed multiple biologic medicines. Post‐market studies should be prioritised to monitor safety among these groups.

## Summary

- The prevalence of use of biologic medicines for paediatric immune‐mediated inflammatory diseases in Australia rose consistently from 2014 to 2020, suggesting children were remaining on treatment over extended periods.
- Over half of the children were first dispensed biologic medicines for inflammatory bowel diseases, one third for inflammatory arthropathies and one in ten for inflammatory skin diseases.
- Infliximab was the most frequently initiated biologic medicine, followed by adalimumab and etanercept.
- Mean age at initiation was 14 years, likely reflecting adherence to age‐specific prescribing restrictions and treatment eligibility criteria.
- Nearly one in five children were dispensed two or more unique biologic medicines during the study period.

## Plain Language Summary

In this study, we examined how often biologic medicines were used by children in Australia to treat immune‐mediated inflammatory diseases and described the characteristics of those starting treatment. Using national medicine dispensing data, we found that the use of these medicines increased between 2014 and 2020, suggesting that many children stayed on treatment for long periods. The most commonly treated condition was inflammatory bowel disease. Older children were more likely to start biologic treatment than younger children, likely due to clinicians following aged‐based prescribing guidelines. A small number of children may have been using biologic medicines off‐label. While most children were dispensed only one biologic medicine, some were dispensed two or more during the study period. Although some of these medicines have been available for over a decade, there is still limited knowledge about their safety in children. Future research should focus on children using multiple biologic medicines or taking them over long periods to better understand how safe and effective these treatments really are.

## 1 Introduction

Paediatric immune‐mediated inflammatory diseases (IMIDs) are a clinically diverse group of conditions characterised by chronic systemic inflammation affecting various body systems in children [1, 2, 3]. Global incidence of IMIDs in the broader population has grown over time [4]. These conditions significantly impact the quality of life, particularly for children transitioning from family and growth‐centred paediatric care to independent adult life [5].

Historically, IMIDs were managed with conventional immunomodulatory medicines such as azathioprine and methotrexate. These medicines are moderately effective but are associated with poor adherence and tolerance due to side effects [6]. Over the past two decades, there has been an exponential increase in the number of biologic medicines introduced to treat IMIDs, which have demonstrated clinical efficacy against placebo or conventional therapy [7, 8, 9]. Currently, there are 10 biologic medicines approved by the Therapeutic Goods Administration (TGA) to treat paediatric IMIDs, including inflammatory arthropathies, inflammatory bowel diseases (IBD), inflammatory skin diseases and severe asthma [8, 10].

Safety knowledge of biologic medicines relies mostly on clinical trials, which often exclude the paediatric population due to ethical considerations, challenges in obtaining informed consent, and the complexities inherent in paediatric clinical research [11, 12]. Consequently, important safety information is often missing for these younger populations at market entry. Additionally, post‐market safety studies largely focus on adult IMID populations [7, 13]. While paediatric safety studies on biologic therapies exist, they mainly focus on inflammatory arthropathies and IBD [14, 15]. To date, only one international study has specifically examined the use of biologic medicines for paediatric IMIDs [16], and no similar study has been conducted in Australia. Understanding the extent of use and the characteristics of children using biologic medicines in the real‐world can help prioritise post‐market safety studies in this population. Therefore, we aim to (1) quantify the uptake and use of biologic medicines for paediatric IMIDs and (2) characterise the Australian paediatric population initiating biologic medicines for IMIDs.

## 2 Methods

### 2.1 Study Setting and Data Source

Australia maintains a publicly funded, universal healthcare system that entitles all citizens and permanent residents to subsidised medicines through the national Pharmaceutical Benefits Scheme (PBS) [17]. Private health insurance does not provide reimbursement for PBS‐subsidised prescription medicines and patients are unlikely to access medicines through unsubsidised avenues (e.g., by paying out of pocket).

PBS dispensing claims are maintained by the Department of Health, Disability and Aging and processed by Services Australia [18, 19]. These data capture PBS‐listed medicines dispensed in community pharmacies, private hospitals, and on discharge from most public hospitals. The PBS data include patient demographic information (sex and year of birth), as well as details of the medicines dispensed (PBS‐specific medicine code denoting formulation, treatment indication, date of dispensing, and the quantity dispensed). PBS data do not capture medicines dispensed to public hospital inpatients, discharge medicines from public hospitals in New South Wales, or private (self‐funded) dispensings.

In this study, we used PBS dispensing claims from two random samples of the PBS‐eligible Australian population aged under 25 years. The data were supplied in two time periods: (1) 1 February 2013 and 30 June 2017, and (2) 1 July 2017 and 31 December 2020. For the first period, Services Australia extracted dispensing records for a 15% random sample of PBS‐eligible residents under 25 years. For the second period, Services Australia extracted dispensing records for the same random 15% sample as the first period and extracted dispensing records for an additional random 15% sample of the PBS‐eligible population under 25 years. Our analysis dataset, therefore, contained dispensing records for a random 15% sample of the PBS‐eligible population from 1 February 2013 to 31 December 2020, as well as records for an additional random 15% sample from 1 July 2017 to 31 December 2020.

### 2.2 Study Design and Population

Our population‐based study comprised children aged 1–17 years dispensed at least one biologic medicine of interest during the study period.

### 2.3 Conditions and Medicines of Interest

We examined biologic medicines approved by the TGA and listed on the PBS for paediatric use in inflammatory arthropathies (juvenile idiopathic arthritis (JIA), psoriatic arthritis), IBD (Crohn's disease, ulcerative colitis), inflammatory skin conditions (chronic plaque psoriasis, chronic spontaneous urticaria, hidradenitis suppurativa), and severe asthma; or medicines not specifically PBS‐listed for paediatric use but potentially used in paediatric populations (Table 1; Figure S1). We searched the TGA and PBS websites and reviewed all publicly available documents for medicines listed for each condition of interest [20]. Although adalimumab and infliximab are currently listed on the PBS for adult use only in ankylosing spondylitis, the TGA–approved product information include an indication for ankylosing spondylitis without specifying whether it applies to adults or children. As this indication appears to be generalised to both adults and children, we included adalimumab and infliximab in our analysis for paediatric ankylosing spondylitis. Similarly, we included ustekinumab in our analysis for paediatric IBD; while not being TGA‐approved or PBS‐listed for this condition, we identified specific PBS listings (PBS medicine codes) for complex fistulising Crohn's disease that do not specify an age restriction and we included ustekinumab for this indication. Ultimately, we identified a total of nine medicines of interest: adalimumab, benralizumab, etanercept, infliximab, mepolizumab, omalizumab, secukinumab, tocilizumab, and ustekinumab. We excluded dupilumab, as it was only PBS‐listed for paediatric use in 2021, which was outside the study period. In the context of this study, we refer to these medicines as IMIDs biologic medicines or medicines of interest.

**TABLE 1:** Biologic medicines approved by the TGA and included in the PBS for paediatric IMIDs.

Biologic medicine	Medicine class	Inflammatory arthropathies			Inflammatory bowel diseases		Inflammatory skin diseases			Severe asthma
		Ankylosing spondylitis	Juvenile idiopathic arthritis	Juvenile psoriatic arthritis	Crohn's disease	Ulcerative colitis	Chronic plaque psoriasis	Chronic spontaneous urticaria	Hidradenitis suppurativa	
Adalimumab	TNF‐α inhibitor	*	✓	—	✓	✓	#	—	✓	—
Etanercept	TNF‐α inhibitor	—	✓	—	—	—	✓	—	—	—
Infliximab	TNF‐α inhibitor	*	—	—	✓	✓	—	—	—	—
Secukinumab	IL‐17A inhibitor	—	#	#	—	—	—	—	—	—
Tocilizumab	IL‐6 inhibitor	—	✓	—	—	—	—	—	—	—
Ustekinumab	IL‐12/23 inhibitor	—	—	—	+	—	✓	—	—	—
Benralizumab	Anti‐IL‐5 receptor monoclonal antibody	—	—	—	—	—	—	—	—	✓
Mepolizumab	Anti‐IL‐5 monoclonal antibody	—	—	—	—	—	—	—	—	✓
Omalizumab	Anti‐IgE monoclonal antibody	—	—	—	—	—	—	✓	—	✓

*Note:* ✓TGA‐approved and PBS‐listed for paediatric use. –#TGA‐approved for paediatric use, PBS‐listed for adult use only. *Age restriction not available in TGA‐approved product information; PBS‐listed for adult use only. +TGA‐approved for adult use only, PBS‐listed without age restriction. —Neither TGA‐approved nor PBS‐listed for paediatric use.

Abbreviations: IgE, immunoglobulin E; IL‐inhibitor, interleukin inhibitor; IMIDs, immune‐mediated inflammatory diseases; PBS, Pharmaceutical Benefits Scheme; TGA, Therapeutic Goods Administration; TNF‐α inhibitor, tumour necrosis factor alpha inhibitor.

### 2.4 Outcomes and Statistical Analyses

#### 2.4.1 Incidence and Prevalence of Use of IMIDs Biologic Medicines

We calculated the incidence of IMIDs biologic medicine use as the number of people dispensed each medicine of interest during each year from 2014 through 2020 with no dispensings of that specific medicine during the preceding 12 months (numerator). We calculated prevalence of IMIDs biologic medicine use as the number of people with at least one dispensing of each medicine during each year from 2014 through 2020 (numerator). We used the Australian Bureau of Statistics's mid‐year population estimates for 1‐ to 17‐year‐olds as the denominator for both measures and presented estimates per 1 000 000 child residents [21]. We further stratified incidence and prevalence estimates by treatment indication.

The increase in sample size due to the data update in mid‐2017 created a situation of multiple population denominators for 2017 (15% population for January through June, 30% population thereafter), as well as a large group of people for whom incidence cannot be estimated due to a lack of data for the 12 months preceding 2017 (the new 15% sample added to the existing 15% sample). To avoid spurious results due to this artefact of the data, we did not include the incidence and prevalence estimates for 2017.

#### 2.4.2 Cohort and Treatment Characteristics

We used descriptive statistics to summarise patient age, sex and geographic location at the time of first dispensing of any medicine of interest. We grouped age as follows: 1–5 years, 6–11 years, and 12–17 years. These categories are commonly used in Australia [22, 23], and align with the PBS criteria for accessing the medicines of interest in each treatment indication [10]. We counted the number of unique biologic medicines dispensed to each child in the cohort and summarised the proportion of the cohort that was dispensed one, two, or three or more medicines of interest during the study period. We calculated the time between each child's first and last dispensing of any biologic medicine of interest, regardless of any breaks in treatment, as an indication of treatment duration with IMIDs biologic medicines of interest.

All analyses were performed using R version 4.3.3. Due to ethical considerations, we suppressed counts less than five as well as any counts that would allow for a count of less than five to be inferred.

## 3 Results

### 3.1 Incidence and Prevalence of Use of IMIDs Biologic Medicines

**FIGURE 1:**
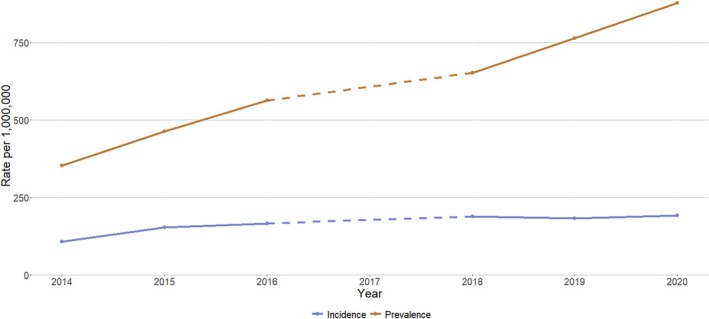
Overall incidence and prevalence of IMIDs biologic medicine use per one million paediatric population, 2014–2020.

The overall incidence of IMIDs biologic medicine use increased from 107.9/1000000 in 2014 to 192.3/1000000 in 2020; prevalence increased from 352.2/1000000 in 2014 to 877.3/1000000 in 2020 over the study period (Figure 1, Table S1).

**FIGURE 2:**
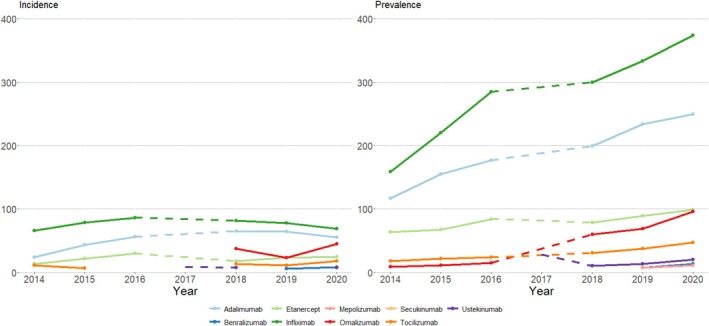
Incidence and prevalence of IMIDs biologic medicine use per one million paediatric population by medicine, 2014–2020.

In 2020, infliximab was the most commonly initiated medicine of interest (44%), followed by adalimumab (27%), and etanercept (12%; Figure 2). The incidence of use of infliximab, adalimumab and etanercept was 66.0, 17.6 and 13.2/1000000, respectively, in 2014, and increased steadily to 88.4, 49.6 and 19.4/1000000, respectively in 2016 (Figure 2, Table S2). Between 2018 and 2020, the incidence declined for infliximab (from 81.7 to 66.5/1000000), remained stable for adalimumab (45.1–44.7/1000000), and increased for etanercept (from 15.7 to 22.9/1000000). From 2014 to 2016, the incidence of use of omalizumab was < 5.0/1000000, rising to 37.7/1000000 in 2018 and peaking at 41.6/1000000 in 2020. Between 2014 and 2020, prevalence of use continued to rise for all IMIDs biologic medicines.

**FIGURE 3:**
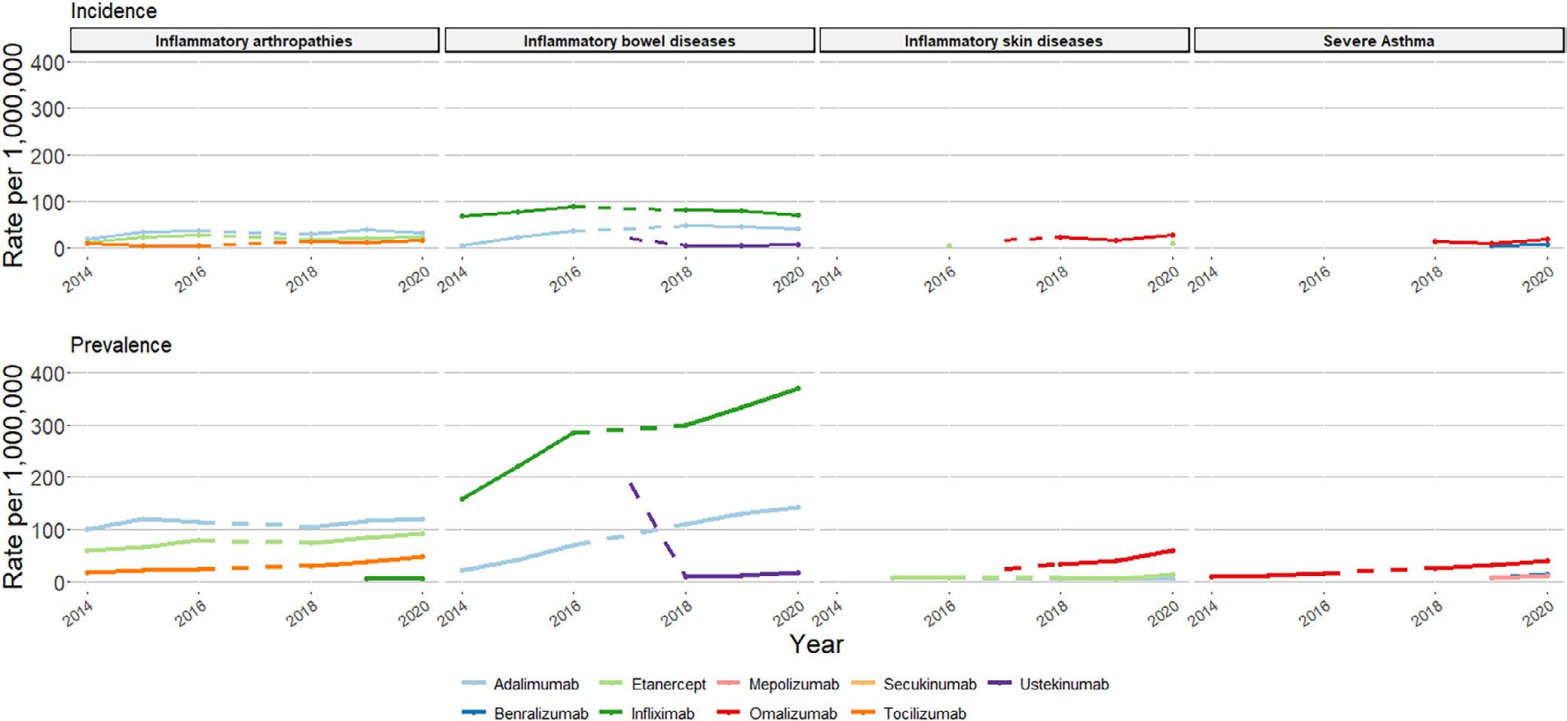
Incidence and prevalence of IMIDs biologic medicine use per one million paediatric population by medicine and indication, 2014–2020.

For paediatric inflammatory arthropathies, adalimumab was the most commonly initiated medicine, increasing from 19.8/1000000 in 2014 32.2/1000000 in 2020. Prevalence rose from 99.1 to 119.5/1000000 during the same period. Etanercept was the second‐most commonly initiated medicine of interest for this indication, followed by tocilizumab. Although infliximab is not PBS‐listed for inflammatory arthropathies, its prevalence in treating this indication increased slightly from < 5.0/1000000 in 2018 to 6.2/1000000 in 2020. Infliximab was most frequently initiated for paediatric IBD, increasing from 68.2/1000000 at its PBS listing for paediatric ulcerative colitis in 2014 to 88.4/1000000 in 2016. While the infliximab incidence declined between 2018 and 2020, prevalence continued to rise over the same period (Figure 3). A similar trend was observed for adalimumab, the second most frequently dispensed medicine of interest for paediatric IBD. Adalimumab was first subsidised for paediatric Crohn's Disease in 2015 and ulcerative colitis in 2016 and incidence of its use increased from 24.0/1000000 in 2015 to 41.6/1000000 in 2020, while prevalence increased from 41.5 to 141.4/1000000 over the same period. Few children initiated ustekinumab, with incidence rising from 5.2/1000000 in 2018 to 7.3/1000000 in 2020.

Omalizumab (PBS‐listed in 2017 for chronic spontaneous urticaria) was more frequently initiated for inflammatory skin diseases compared to etanercept (PBS‐listed in 2012 for chronic plaque psoriasis), with both incidence and prevalence showing one‐ and two‐fold increases, respectively, over time (incidence: 24.1/1000000 in 2018 to 29.1/1000000 in 2020; prevalence: 33.5/1000000 in 2018 to 60.3/1000000 in 2020). For severe asthma, omalizumab (PBS‐listed in 2011 for severe allergic asthma) was also more commonly initiated than benralizumab and mepolizumab (PBS‐listed in 2017 and 2018, respectively). While the incidence and prevalence of omalizumab use in severe asthma were low at the beginning of study period, both increased more than fourfold over time (incidence: < 5/1000000 in 2014 to 20/1000000 in 2020; prevalence: 9/1000000 to 41/1000000 over the same period).

### 3.2 Cohort and Treatment Characteristics

We identified 1131 Australian children (51% male) dispensed at least one of the nine medicines of interest over the study period (Table 2). At initiation of first medicine of interest, mean age was 13.7 years (standard deviation: 3.5 years). Most children were aged 12–17 years (82%) and resided in a major Australian city (73%; Table S4). The most common indications for which children initiated their first medicine of interest were IBD (55%), inflammatory arthropathies (29%), inflammatory skin diseases (10%), and severe asthma (6%).

**TABLE 2:** Characteristics of Australian children initiating biologic medicines for IMIDs.

Demographics	Overall
*n* (%)	1131 (100)
Sex, *n* (%)	
Male	572 (51)
Age, median (range), years	14.3 (12.1–16.5)
Age groups, *n* (%)	
1–5 years	33 (3)
6–11 years	175 (15)
12–17 years	923 (82)
Geographic location, *n* (%)	
Major cities	821 (73)
Regional areas	287 (25)
Remote or very remote areas	18 (2)
Unknown	5 (< 1)

We observed 209 (18%) children dispensed two or more unique medicines of interest for any IMID condition during the study period. Specifically, 177 (15%) were dispensed two unique biologic medicines, and 34 (3%) were dispensed three or more unique medicines of interest.

The median time between first and last dispensings of any biologic medicine of interest over the study period was 2.53 years (IQR: 1.07–3.71 years; Table S7). Children dispensed infliximab had a median treatment duration of 2.31 years (IQR: 0.87–3.65 years), while median treatment durations associated with adalimumab and etanercept were 1.84 years (IQR: 0.71–3.32 years) and 1.78 years (IQR: 0.58–3.52 years), respectively.

## 4 Discussion

Our study found that while the incidence of IMIDs biologic medicine use increased over time, the prevalence rose by nearly 2.5‐fold, suggesting that children were potentially remaining on these treatments (median duration of 2.53 years). The median age was 14 years, and nearly one in five were dispensed two or more unique biologic medicines during the study period. To our knowledge, these are the first national, population‐based estimates providing an overview of contemporary biologic medicine use for IMIDs in Australian children.

Our findings of a steady increase in uptake align with previous studies that have reported a consistent rise in the incident use of biologic therapies in paediatric IBD and JIA [24, 25]. The introduction of biosimilars and the expansion of PBS indications may have contributed to the gradual increase in uptake we observed. Nevertheless, prescribers in Australia are required to adhere to the PBS prescribing criteria. In standard clinical practice, conventional therapies (e.g., azathioprine and methotrexate) are commonly prescribed as first‐ or second‐line treatment before biologic treatments are considered [10]. The consistent increase in uptake in our study may reflect increasing prescriber confidence despite limited safety information.

The growing prevalence of use in our study is consistent with the previous research examining biologic exposure in paediatric IBD and JIA [24, 25]. Our findings demonstrate that most children remained on treatment, aligning with prior studies reporting continued prescribing of these medicines in patients who show positive treatment responses and maintain remission without adverse outcomes [26, 27]. However, concerns about disease relapse may contribute to hesitancy among prescribers to discontinue treatment, even when clinical remission is achieved [28]. These uncertainties raise important questions about the long‐term management and safety of biologic therapies in children, including the overall benefit–risk balance of continued therapy.

Infliximab was the most commonly initiated medicine for paediatric IBD. Although adalimumab is also effective and approved for this indication, it remained the second most frequently used. This finding aligns with several international studies that have similarly reported higher use of infliximab compared to adalimumab in paediatric IBD populations [24, 29, 30]. There are no robust head‐to‐head trials directly comparing infliximab and adalimumab, but both medicines have demonstrated comparable safety and effectiveness in observational studies [31, 32, 33]. The higher use of infliximab throughout our study may reflect its earlier PBS listing for paediatric Crohn's Disease in 2007. Prescriber confidence and experience with infliximab may have influenced treatment decisions, particularly given its intravenous mode of administration in a clinical setting that allows for structured monitoring.

The wider use of adalimumab for JIA in our study may be attributed to its less frequent dosing schedule, which is administered fortnightly, compared to the weekly etanercept dosing [34, 35]. Another contributing factor may be adalimumab's efficacy in treating anterior uveitis, a common complication of JIA [36]. However, our findings differ from previous studies that reported higher use of etanercept in JIA [37, 38, 39]. This discrepancy may be due to etanercept's earlier approval in the US (1999 vs. 2008 for adalimumab), and its relatively painless injection experience, compared to earlier citrate‐containing formulations of adalimumab, which were associated with greater injection site pain [25]. Our results are more consistent with a recent study indicating that adalimumab became the dominant treatment after 2018, likely due to the introduction of improved, citrate‐free formulations that reduce injection site pain [25]. Previous research has reported an increase in tocilizumab use in children, particularly those with systemic JIA, highlighting better compliance and survival rates with early initiation [38, 40, 41]. However, the lower tocilizumab use in our study suggests that prescribing patterns may differ. Further Australian‐based studies are needed to investigate the factors influencing these trends, including prescriber preferences, clinical considerations and potential barriers to tocilizumab uptake, including potential adverse outcomes.

Our study observed a higher uptake of omalizumab for severe asthma from 2018, compared to benralizumab or mepolizumab. This may be due to omalizumab's established clinical efficacy [42, 43], and the later introduction of benralizumab and mepolizumab with limited safety data available for paediatric use. However, despite concerns about paediatric malignancies [44, 45, 46, 47, 48], the uptake and use of omalizumab rose throughout the study period, suggesting a need for further investigation into its adverse event profile and longer‐term safety.

We identified a small proportion of children dispensed infliximab for inflammatory arthropathies outside PBS criteria. While the TGA‐approved product information does not specify an age restriction and infliximab has been approved for juvenile ankylosing spondylitis in Europe [49], there is a lack of established clinical guidance for paediatric dosing regimens. Similarly, ustekinumab is not TGA‐approved for paediatric IBD, yet we observed a gradual increase in use for this indication over the study period. This may be driven by broader prescribing practices, potentially through the selection of certain PBS codes that do not specify age criteria or through off‐label use informed by international literature. Future studies should evaluate off‐label use for these indications to provide more robust safety evidence that can guide clinical decision making.

We found that IMIDs biologic medicines were predominantly initiated in older children, as they were more likely to meet clinical and PBS criteria for treatment eligibility. Our findings of some children initiating multiple IMIDs biologic medicines align with a previous study that reported 7.2% of children had received four or more biologic medicines for JIA [38]. However, we examined the number of unique medicines dispensed throughout the study period and did not investigate concomitant use. Clinical guidelines generally do not recommend concomitant use, and there are no specific guidelines outlining sequential biologic treatment. With multiple effective treatment options available, identifying the most optimal first‐line biologic therapy remains uncertain and challenging, which may explain why some children in our study were dispensed two or more unique medicines, potentially due to treatment switching or use for different indications.

Our estimates of treatment duration were crude, calculated as the difference between first and last dispensing. Taken with our findings that prevalence rates outpace incidence rates, these results further suggest that children continue treatment for extended periods. Nevertheless, specific recommendations for treatment duration are often lacking in clinical guidelines, which can lead to variability in prescribing practices and potentially impact the long‐term safety in children receiving biologic treatment. More detailed investigations are warranted to determine the optimal treatment duration, and to identify when, and if, children should safely discontinue biologic treatment.

### 4.1 Strengths and limitations

Our study used patient‐level data from a large, population‐based sample to quantify the uptake of biologic medicines in Australian children. Our findings are generalisable to similar developed nations' populations and health systems. However, our data did not include information on the prescribed dose or actual frequency of treatments. Further, as with all administrative dispensing data, we did not observe whether the medicines dispensed were ultimately administered to children. Our data did not include diagnosis information, and we ascertained treatment indication from PBS specific codes, which might not accurately reflect the underlying diagnoses and could be subject to error. We were unable to determine the proportion of children with a specific condition who received treatment for that condition as population denominators were unavailable.

## 5 Conclusions

Children were remaining on IMIDs biologic medicines for extended periods, yet despite most medicines having been available for over a decade, their long‐term safety and adverse event profiles in children remain poorly understood. Initiated biologic treatment for inflammatory bowel diseases was most common, however a small proportion of children were dispensed biologic medicines not approved for paediatric IMIDs, indicating potential off‐label use. Future studies should prioritise investigating long‐term safety, multiple use, and off‐label use of biologic medicines in paediatric IMIDs.

## Author Contributions

J.N.H., B.D. and N.P. created the initial concept and study design. J.N.H. and B.D. analysed the data. J.N.H. wrote the original draft and revised subsequent versions. T.B., H.Z., N.N., J.B.H., N.P., and B.D. provided feedback and contributed to manuscript editing. All authors critically reviewed, revised and approved the final draft.

## Funding

This study was supported by Australian National Health and Medical Research Council (NHMRC; GNT1157506) and NHMRC Centre of Research Excellence in Medicines Intelligence (GNT1196900). J.N.H. is supported by a University of South Australia President's Scholarship. N.N. is supported by NHMRC Investigator Grant (APP1197940) and Financial Markets Foundation for Children. B.D. is supported by a Cancer Institute NSW Early Career Fellowship (ECF1381).

## Ethics Statement

This study was approved by the New South Wales Population and Health Services Research Ethics Committee Executive Committee (2019/ETH01776—2023UMB0806).

## Conflicts of Interest

T.B. received payment from the University of California, Berkeley, for participation in a Data Safety Monitoring Board for the treatment of juvenile idiopathic arthritis. N.P. is a member of the Drug Utilisation Sub Committee of the Pharmaceutical Benefits Advisory Committee. The other authors declare no conflicts of interest that are relevant to the content of this article.

## Supporting information


**Figure S1:** Timeline of biologic medicines listed on the Australian Pharmaceutical Benefits Scheme by paediatric IMID indication.
**Table S1:** Overall incidence and prevalence of IMIDs biologic medicine use per one million paediatric population, 2014–2020.
**Table S2:** Incidence and prevalence of IMIDs biologic medicine use per one million paediatric population by medicine, 2014–2020.
**Table S3:** Incidence and prevalence of IMIDs biologic medicine use per one million paediatric population by medicine and indication, 2014–2020.
**Table S4:** Age of paediatric patients initiating IMIDs biologic medicines.
**Table S5:** Time between first and last dispensing of IMIDs biologic medicines during study period.

## Data Availability

The data that supports the findings of this study are available in the Supporting Information of this article.
