# Supplementary material for: Uptake and Use of Biologic Therapies in Paediatric Immune‐Mediated Inflammatory Diseases: An Australian Population‐Based Study
Source: Pharmacoepidemiol Drug Saf. 2026 Jul 1;35(7):e70412. doi: 10.1002/pds.70412 (PMC13322984; doi:10.1002/pds.70412)
Supplement: Supplementary file 1 — Figure S1: Timeline of biologic medicines listed on the Australian Pharmaceutical Benefits Scheme by paediatric IMID indication. Table S1: Overall incidence and prevalence of IMIDs biologic medicine use per one million paediatric population, 2014–2020. Table S2: Incidence and prevalence of IMIDs biologic medicine use per one million paediatric population by medicine, 2014–2020. Table S3: Incidence and prevalence of IMIDs biologic medicine use per one million paediatric population by medicine and indication, 2014–2020. Table S4: Age of paediatric patients initiating IMIDs biologic medicines. Table S5: Time between first and last dispensing of IMIDs biologic medicines during study period. [file PDS-35-e70412-s001.docx]

**Supporting Information**

Uptake and use of biologic therapies in paediatric immune-mediated inflammatory diseases: an Australian population-based study

**Table of Contents**

**Figure S1.** Timeline of biologic medicines listed on the Australian Pharmaceutical Benefits Scheme by paediatric IMID indication

**Table S1.** Overall incidence and prevalence of IMIDs biologic medicine use per one million paediatric population, 2014–2020

**Table S2.** Incidence and prevalence of IMIDs biologic medicine use per one million paediatric population by medicine, 2014–2020

**Table S3.** Incidence and prevalence of IMIDs biologic medicine use per one million paediatric population by medicine and indication, 2014–2020

**Table S4.** Age of paediatric patients initiating IMIDs biologic medicines

**Table S5.** Time between first and last dispensing of IMIDs biologic medicines during study period

**Figure S1.** Timeline of biologic medicines listed on the Australian Pharmaceutical Benefits Scheme by paediatric IMID indication


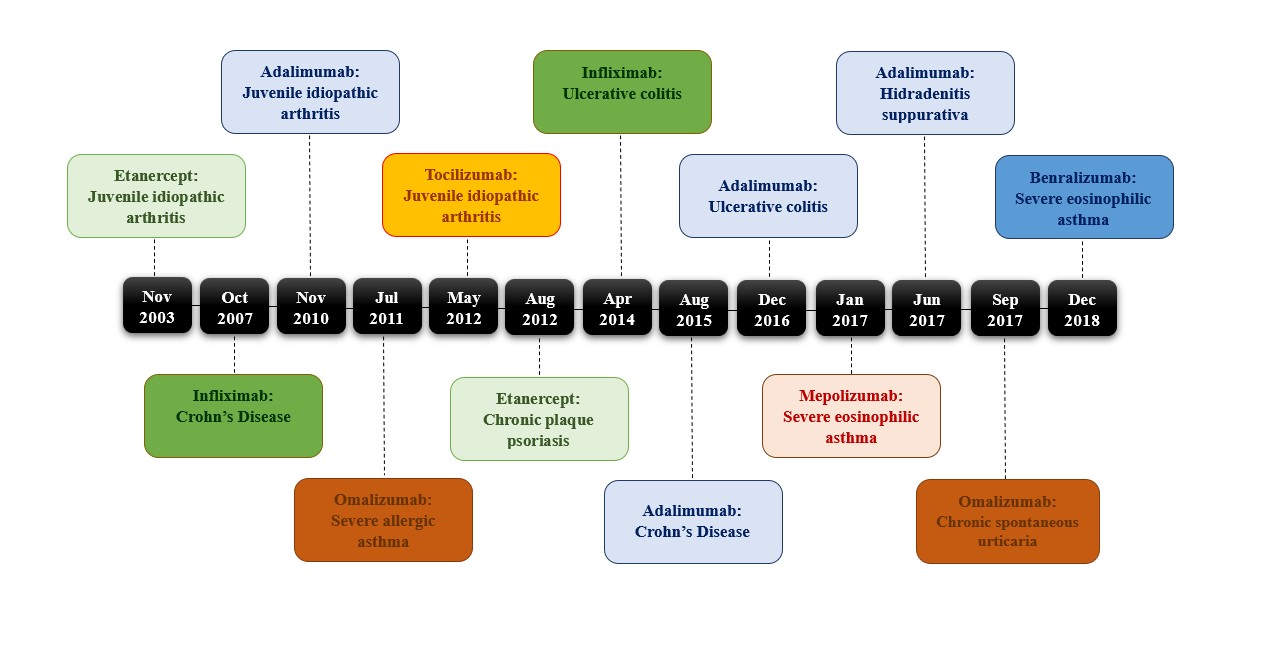


**Table S1.** Overall incidence and prevalence of IMIDs biologic medicine use per one million paediatric population, 2014–2020

| Year | Incidence | Prevalence |
| --- | --- | --- |
| 2014 | 107.9 | 352.2 |
| 2015 | 152.7 | 462.5 |
| 2016 | 166.0 | 562.8 |
| 2017 | * | * |
| 2018 | 188.7 | 651.9 |
| 2019 | 182.9 | 763.9 |
| 2020 | 192.3 | 877.3 |

*Incidence and prevalence estimates for 2017 not included due to artefact of the data (refer to Methods)

**Table S2.** Incidence and prevalence of IMIDs biologic medicine use per one million paediatric population by medicine, 2014–2020

| Biologic medicine | Year | Incidence | Prevalence |
| --- | --- | --- | --- |
| Adalimumab | 2014 | 17.6 | 116.7 |
|  | 2015 | 45.8 | 154.9 |
|  | 2016 | 49.6 | 176.8 |
|  | 2017 | * | * |
|  | 2018 | 45.1 | 199.1 |
|  | 2019 | 44.7 | 233.8 |
|  | 2020 | 44.7 | 249.5 |
| Benralizumab | 2018 | <5.0 | <5.0 |
|  | 2019 | 5.2 | 7.3 |
|  | 2020 | <5.0 | 13.5 |
| Etanercept | 2014 | 13.2 | 63.8 |
|  | 2015 | 17.5 | 67.6 |
|  | 2016 | 19.4 | 84.1 |
|  | 2017 | * | * |
|  | 2018 | 15.7 | 78.6 |
|  | 2019 | 22.9 | 89.4 |
|  | 2020 | 22.9 | 98.8 |
| Infliximab | 2014 | 66.0 | 158.5 |
|  | 2015 | 78.5 | 220.4 |
|  | 2016 | 88.4 | 284.7 |
|  | 2017 | * | * |
|  | 2018 | 81.7 | 299.7 |
|  | 2019 | 75.9 | 333.6 |
|  | 2020 | 66.5 | 374.2 |
| Mepolizumab | 2017 | * | * |
|  | 2018 | <5.0 | <5.0 |
|  | 2019 | <5.0 | 7.3 |
|  | 2020 | <5.0 | 11.4 |

Table S2 (cont.)

| Biologic medicine | Year | Incidence | Prevalence |
| --- | --- | --- | --- |
| Omalizumab | 2014 | <5.0 | 8.8 |
|  | 2015 | <5.0 | 10.9 |
|  | 2016 | <5.0 | 15.1 |
|  | 2017 | * | * |
|  | 2018 | 37.7 | 59.7 |
|  | 2019 | 24.9 | 68.6 |
|  | 2020 | 41.6 | 95.6 |
| Secukinumab | 2016 | <5.0 | <5.0 |
|  | 2017 | * | * |
|  | 2018 | <5.0 | <5.0 |
|  | 2019 | <5.0 | <5.0 |
|  | 2020 | <5.0 | <5.0 |
| Tocilizumab | 2014 | 6.6 | 17.6 |
|  | 2015 | 6.5 | 21.8 |
|  | 2016 | <5.0 | 23.7 |
|  | 2017 | * | * |
|  | 2018 | 6.3 | 30.4 |
|  | 2019 | 5.2 | 37.4 |
|  | 2020 | 8.3 | 46.8 |
| Ustekinumab | 2018 | <5.0 | 10.5 |
|  | 2019 | <5.0 | 13.5 |
|  | 2020 | <5.0 | 19.8 |

*Incidence and prevalence estimates for 2017 not included due to artefact of the data (refer to Methods)

**Table S3.** Incidence and prevalence of IMIDs biologic medicine use per one million paediatric population by medicine and indication, 2014–2020

| Indication | Biologic medicine | Year | Incidence | Prevalence |
| --- | --- | --- | --- | --- |
| Inflammatory arthropathies | Adalimumab | 2014 | 19.8 | 99.1 |
|  |  | 2015 | 34.9 | 120.0 |
|  |  | 2016 | 36.7 | 114.3 |
|  |  | 2017 | * | * |
|  |  | 2018 | 30.4 | 103.8 |
|  |  | 2019 | 38.5 | 116.4 |
|  |  | 2020 | 32.2 | 119.5 |
|  | Etanercept | 2014 | 13.2 | 59.4 |
|  |  | 2015 | 24.0 | 65.5 |
|  |  | 2016 | 28.0 | 79.8 |
|  |  | 2017 | * | * |
|  |  | 2018 | 18.9 | 73.4 |
|  |  | 2019 | 20.8 | 83.1 |
|  |  | 2020 | 22.9 | 91.5 |
|  | Infliximab | 2017 | * | * |
|  |  | 2018 | <5.0 | <5.0 |
|  |  | 2019 | <5.0 | 5.2 |
|  |  | 2020 | <5.0 | 6.2 |
|  | Secukinumab | 2018 | <5.0 | <5.0 |
|  |  | 2019 | <5.0 | <5.0 |
|  |  | 2020 | <5.0 | <5.0 |
|  | Tocilizumab | 2014 | 11.0 | 17.6 |
|  |  | 2015 | 6.5 | 21.8 |
|  |  | 2016 | 6.5 | 23.7 |
|  |  | 2017 | * | * |
|  |  | 2018 | 13.6 | 30.4 |
|  |  | 2019 | 11.4 | 37.4 |
|  |  | 2020 | 17.7 | 46.8 |
|  | Ustekinumab | 2020 | <5.0 | <5.0 |

Table S3 (cont.)

| Indication | Biologic medicine | Year | Incidence | Prevalence |
| --- | --- | --- | --- | --- |
| Inflammatory bowel diseases | Adalimumab | 2014 | 6.6 | 22.0 |
|  |  | 2015 | 24.0 | 41.5 |
|  |  | 2016 | 36.7 | 69.0 |
|  |  | 2017 | * | * |
|  |  | 2018 | 48.2 | 110.0 |
|  |  | 2019 | 45.7 | 130.9 |
|  |  | 2020 | 41.6 | 141.4 |
|  | Infliximab | 2014 | 68.2 | 158.5 |
|  |  | 2015 | 78.5 | 220.4 |
|  |  | 2016 | 88.4 | 284.7 |
|  |  | 2017 | * | * |
|  |  | 2018 | 81.7 | 298.7 |
|  |  | 2019 | 79.0 | 332.6 |
|  |  | 2020 | 70.7 | 370.1 |
|  | Ustekinumab | 2017 | * | * |
|  |  | 2018 | 5.2 | 8.4 |
|  |  | 2019 | 5.2 | 11.4 |
|  |  | 2020 | 7.3 | 17.7 |

Table S3 (cont.)

| Indication | Biologic medicine | Year | Incidence | Prevalence |
| --- | --- | --- | --- | --- |
| Inflammatory skin diseases | Adalimumab | 2015 | <5.0 | <5.0 |
|  |  | 2016 | <5.0 | 6.5 |
|  |  | 2017 | * | * |
|  |  | 2018 | <5.0 | 5.2 |
|  |  | 2019 | <5.0 | 6.2 |
|  |  | 2020 | <5.0 | 5.2 |
|  | Etanercept | 2014 | <5.0 | <5.0 |
|  |  | 2015 | <5.0 | 6.5 |
|  |  | 2016 | 6.5 | 6.5 |
|  |  | 2017 | * | * |
|  |  | 2018 | <5.0 | 7.3 |
|  |  | 2019 | <5.0 | 6.2 |
|  |  | 2020 | 10.4 | 12.5 |
|  | Omalizumab | 2017 | * | * |
|  |  | 2018 | 24.1 | 33.5 |
|  |  | 2019 | 17.7 | 40.5 |
|  |  | 2020 | 29.1 | 60.3 |
|  | Secukinumab | 2016 | <5.0 | <5.0 |
|  |  | 2017 | <5.0 | <5.0 |
|  |  | 2018 | <5.0 | <5.0 |
|  |  | 2019 | <5.0 | <5.0 |
|  |  | 2020 | <5.0 | <5.0 |
|  | Ustekinumab | 2018 | <5.0 | <5.0 |
|  |  | 2019 | <5.0 | <5.0 |
|  |  | 2020 | <5.0 | <5.0 |

Table S3 (cont.)

| Indication | Biologic medicine | Year | Incidence | Prevalence |
| --- | --- | --- | --- | --- |
| Severe asthma | Benralizumab | 2018 | <5.0 | <5.0 |
|  |  | 2019 | 6.2 | 7.3 |
|  |  | 2020 | 8.3 | 13.5 |
|  | Mepolizumab | 2017 | * | * |
|  |  | 2018 | <5.0 | <5.0 |
|  |  | 2019 | <5.0 | 7.3 |
|  |  | 2020 | <5.0 | 11.4 |
|  | Omalizumab | 2014 | <5.0 | 8.8 |
|  |  | 2015 | <5.0 | 10.9 |
|  |  | 2016 | <5.0 | 15.1 |
|  |  | 2017 | * | * |
|  |  | 2018 | 13.6 | 26.2 |
|  |  | 2019 | 10.4 | 31.2 |
|  |  | 2020 | 19.8 | 40.5 |

*Incidence and prevalence estimates for 2017 not included due to artefact of the data (refer to Methods)

**Table S4.** Age of paediatric patients initiating IMIDs biologic medicines

| Indication | Biologic medicine | Age group (years) | n (%) |
| --- | --- | --- | --- |
| Inflammatory arthropathies | Adalimumab | 1-2 | <5 |
|  |  | 3-5 | 8 (5) |
|  |  | 6-11 | 39 (22) |
|  |  | 12-17 | 125 (71) |
|  | Etanercept | 1-2 | <5 |
|  |  | 3-5 | 12 (10) |
|  |  | 6-11 | 28 (24) |
|  |  | 12-17 | 74 (62) |
|  | Secukinumab | 12-17 | <5 |
|  | Tocilizumab | 1-2 | 8 (20) |
|  |  | 3-5 | 9 (22) |
|  |  | 6-11 | 12 (29) |
|  |  | 12-17 | 12 (29) |
| Inflammatory bowel diseases | Adalimumab | 1-2 | <5 |
|  |  | 3-5 | <5 |
|  |  | 6-11 | 17 (13) |
|  |  | 12-17 | 103 (79) |
|  | Infliximab | 3-5 | <5 |
|  |  | 6-11 | 100 (20) |
|  |  | 12-17 | 391 (79) |
| Inflammatory skin diseases | Adalimumab | 12-17 | 12 (100) |
|  | Etanercept | 6-11 | 5 (24) |
|  |  | 12-17 | 16 (76) |
|  | Omalizumab | 1-2 | <5 |
|  |  | 6-11 | 7 (8) |
|  |  | 12-17 | 76 (86) |
| Severe asthma | Benralizumab | 12-17 | 9 (100) |
|  | Mepolizumab | 12-17 | 5 (100) |
|  | Omalizumab | 6-11 | 17 (32) |
|  |  | 12-17 | 36 (68) |

**Table S5.** Time between first and last dispensing of IMIDs biologic medicines during study period

|  | Median time (range), years |
| --- | --- |
| Overall | 2.35 (1.07–3.71) |
| Adalimumab | 1.84 (0.71–3.32) |
| Benralizumab | 0.42 (0.18–1.43) |
| Etanercept | 1.78 (0.58–3.52) |
| Infliximab | 2.31 (0.87–3.65) |
| Mepolizumab | 1.11 (0.58–2.23) |
| Omalizumab | 1.30 (0.56–2.43) |
| Secukinumab | 0.62 (0.22–2.04) |
| Tocilizumab | 1.36 (0.38–2.82) |
| Ustekinumab | 1.10 (0.31–2.55) |
